# Saving a species, one mite at a time: efficacy of afoxolaner (NexGard®) against Macronyssidae mites in the critically endangered Aeolian wall lizard (*Podarcis raffonei*)

**DOI:** 10.1016/j.ijppaw.2026.101245

**Published:** 2026-06-04

**Authors:** Jairo Alfonso Mendoza-Roldan, Alessia Ricci, Benedetta Gambioli, Katerina Sioumpoura, Antonella Di Palma, Gianpaolo Montinaro, Daniele Macale, Leonardo Vignoli, Frederic Beugnet, Domenico Otranto

**Affiliations:** a Department of Veterinary Medicine, University of Bari, Valenzano, Italy; b Department of Sciences, Roma Tre University, Rome, Italy; c Department of Agricultural Sciences, Food, Natural Resources and Engineering, University of Foggia, Foggia, Italy; d RIFCON GmbH, Goldbeckstrasse 13, Hirschberg, Germany; e Fondazione Bioparco di Roma, Rome, Italy; f Parasitology Unit, Faculty of Veterinary Science, Chulalongkorn University, Bangkok, Thailand; g Boehringer Ingelheim Animal Health, Lyon, France; h Department of Veterinary Clinical Sciences, City University of Hong Kong, China

**Keywords:** *Ophionyssus lacertinus*, *Ex-situ* conservation, Isoxazolines, Ectoparasites, *Podarcis siculus*

## Abstract

The Aeolian wall lizard (*Podarcis raffonei*) is an endangered lacertid species endemic to the Aeolian Archipelago (Sicily, Italy). Its populations are threatened by several factors, including habitat fragmentation and reduction, competition with the invasive *Podarcis siculus*, and potential health risks associated with disease dynamics. To prevent the extinction of this unique species an *ex-situ* captive breeding program was established in the Reptile House at the *Bioparco* of Rome within the LIFE project EOLIZARD. Most animals were housed in outdoor enclosures, favoring the exposure of these naïve lizards to new ectoparasitic mite species, that may negatively impact host fitness through direct effects and pathogen transmission. Therefore, given the lack of antiparasitic strategies for small lacertid lizards, this study evaluated the safety and efficacy of oral administration of afoxolaner for controlling mite infestations. A total of 178 lizards were clinically examined and treated with a single oral dose of afoxolaner (2.5 mg/kg). Mite infestation was monitored before treatment (T0) in all lizards. In addition, 20 specimens kept in individual enclosures and 30 from outdoor enclosures, were checked at 24 h (T1), 7 days (T2), 14 days (T3), and 28 days (T4) post-treatment. Ectoparasites were identified using both morphological and molecular methods as *Ophionyssus lacertinus*, and molecular screening was performed to detect vector-borne pathogens. Before treatment, 90 out of 178 lizards (50.6%) were infested with mites, with the prevalence rapidly declining in the follow up, until mite clearance at day 28 post-administration, in single housed lizards, and as low as 5% prevalence in those kept outdoors. Other lizard species (captive and free-living) (i.e., *Podarcis muralis*, *P. siculus*, *Timon lepidus*) were also infested, potentially being the source of mites. These findings suggest that oral afoxolaner is an effective treatment for controlling mite infestations in small lacertid lizards, with important implications for conservation management.

## Highlights

• Efficacy of oral afoxolaner was evaluated in 178 Aeolian wall lizards (*Podarcis raffonei*).
• Ectoparasite prevalence fell from 65% to complete eradication within 28 days under controlled conditions.
• The recovered mites were identified as *Ophionyssus lacertinus* (Macronyssidae).
• Sympatric lizard species may serve as reservoir hosts for these ectoparasites.
• Single oral afoxolaner (2.5 mg/kg) showed high tolerability and efficacy in small lacertid lizards.

## Graphical abstract

**Figure:**
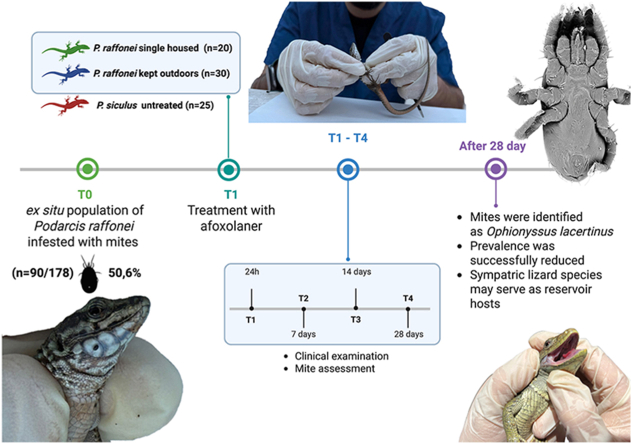

## 1 Introduction

Parasites are increasingly recognized as important drivers of reptile ecology and conservation because they can affect host physiology, behavior, and population dynamics (Bower et al., 2018). In conservation programs targeting threatened species, parasite monitoring is essential for evaluating individual health and preventing pathogen transmission, as shown, for example, in *Glaucomastix abaetensis* lizards (Leite et al., 2025). Similarly, studies on terrestrial chelonians, including *Centrochelys sulcata* and *Chersobius signatus*, have shown that systematic parasite screening and treatment are critical components of effective conservation management (Galosi et al., 2021; Costa et al., 2022).

**Fig. 1:**
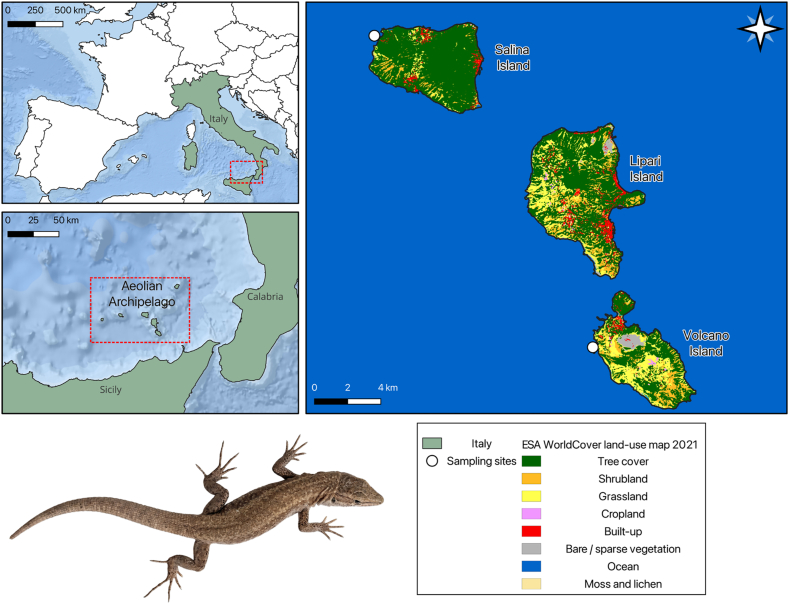
Map of Aeolian Archipelago (Italy) showing specific locations in Salina and Vulcano Islands where *Podarcis raffonei* specimens were collected (white circles). The map was prepared using QGIS software version 3.42 — Maidenhead, with ESA WorldCover land-use map 2021 imagery.

A particularly relevant case is the Aeolian wall lizard, *Podarcis raffonei* (Mertens, 1952), a small lacertid endemic to the Aeolian Archipelago (northern Sicily, Italy). This species exhibits remarkable ecological and morphological adaptations, such as cryptic coloration and high thermal tolerance, which allow it to persist in the harsh, rocky environments of the Aeolian islets. Despite this apparent resilience, *P. raffonei* is listed as Critically Endangered by the IUCN (International Union for Conservation of Nature; IUCN, 2024) because of its extremely small, fragmented, and declining populations, now restricted to only a few tiny localities (i.e., Capo Grosso, Strombolicchio, Scoglio Faraglione, and La Canna; Fig. 1). Currently, the main threats to the persistence of *P. raffonei* are habitat degradation, invasive black rats, and competition/hybridization with the introduced Italian wall lizard (*Podarcis siculus*). The latter has caused severe population declines through ecological competition and genetic introgression resulting from hybridization (Ficetola et al., 2021; Paris et al., 2024). In response to these threats, the LIFE22-NAT-IT-LIFE EOLIZARD (101114121) project aims to halt this decline through targeted conservation measures combining both *ex-situ* and *in-situ* management. The main actions include the establishment of a captive breeding program coordinated by Roma Tre University and the *Fondazione* *Bioparco* of Rome, with the goal of increasing population size under controlled conditions, and the reintroduction of captive-bred individuals into a sanctuary population (Capula et al., 2002; McGowan et al., 2017). Within the Reptile House of the *Bioparco* of Rome founder adults collected from two localities (Scoglio Faraglione and Capo Grosso) are maintained in outdoor enclosures designed to mimic their native island habitats, while preserving pure genetic lineages by keeping the two source populations separate. Eggs laid in these enclosures are transferred to indoor incubators, and hatchlings are subsequently reared in a climate-controlled nursery until they are large enough to be moved outdoors.

However, outdoor housing may increase the risk of exposure to ectoparasites. In Southern Italy, populations of *P. siculus* are frequently infested with ticks (e.g., *Ixodes ricinus*) and mites (e.g., the chigger mite, *Neotrombicula autumnalis*, and the lizard mite *Ophionyssus sauracum*) (Mendoza-Roldan et al., 2021a). Additionally, the snake mite *Ophionyssus natricis* has been found on both *P. siculus* and the insular subspecies *P. s*. *klemmerii*, highlighting the role of this species as a potential reservoir for mites that can affect other reptiles (Dipineto et al., 2018). Beyond their direct effects, such as dermatitis, dysecdysis, anemia, ectoparasitic mites can act as vectors of pathogens, including haemogregarine protozoans (e.g., *Hepatozoon* spp. and *Karyolysus* spp.) and bacterial agents like *Rickettsia* spp., which may impact host health and population fitness (Razmjo et al., 2013; Haklová-Kočíková et al., 2014; Mendoza-Roldan et al., 2021b). Moreover, some of these pathogens are zoonotic and may potentially pose health risks to humans (Mendoza-Roldan et al., 2021c). Additionally, where multiple lacertid species occur in sympatry, ectoparasites may be shared between hosts, facilitating parasite spillover and potentially increasing the parasite burden in vulnerable populations (Fain, 1994). Therefore, given the extremely restricted distribution and small population size of *P. raffonei* particularly at Capo Grosso, where this species co-occurs with the invasive *P. siculus*, even moderate ectoparasite infestations or pathogen exposure could have profound conservation implications (Amo et al., 2005).

Previous studies demonstrated the efficacy of isoxazolines (e.g., afoxolaner) in eradicating mite infestations by *O. natricis* (Macronyssidae) and *Pterygosoma inermis* (Pterygosomatidae), in both captive snakes (Mendoza-Roldan et al., 2023) and medium-to large-sized lizards (Mendoza-Roldan et al., 2025). However, data regarding safety and efficacy of afoxolaner in small lacertid lizards are lacking and the small body size of these animals further complicates pharmacological interventions, as minor inaccuracies in dosing may result in toxicity or subtherapeutic exposure (Bates et al., 2024). Nonetheless, afoxolaner has shown to have a wide safety margin up to 5 times the maximum exposure dose, including repeated dosing in mammals, infrequently presenting mild adverse effects at high multiple doses (i.e., transient vomiting or diarrhea; Drag et al., 2014; Letendre et al., 2014). Therefore, the present study aimed to evaluate the efficacy and tolerability of orally administered afoxolaner in controlling mite infestations in a captive population of *P. raffonei*, as well as to perform clinical follow-up to assess treatment outcomes.

## 2 Material and methods

### 2.1 Ethic statement

All protocols for the handling of reptiles and sample collection were approved by the Committee for Bioethics and Animal Welfare of the Department of Veterinary Medicine, University of Bari, Italy (approval number: 58/24). The administration of afoxolaner was conducted off label, in accordance with the European cascade principle, which allows the use of a veterinary product authorized for a different species when no registered formulation exists for the specific indication in the target species.

### 2.2 Animal screening

The study was conducted on an *ex-situ* breeding population of *P. raffonei* originating from Capo Grosso (40°42′00″N, 13°55′00″E) and Scoglio Faraglione (38°34′46″N, 14°48′02″E) islets, currently housed at the *Bioparco* of Rome (41°55′02.9″N, 12°29′06.7″E). Lizards were housed in large cement enclosures filled with soil and substrate to simulate their natural habitat, including shelters, rocks, and vegetation. Individuals were divided according to their geographic origin, with separate enclosures for animals from Capo Grosso and Scoglio Faraglione, allowing the observation of potential differences in health status, parasite burden, and treatment response.

Prior to the start of the study, all individuals (*n* = 178) underwent a thorough clinical examination, and both the lizards and their enclosures were carefully checked for mite infestations. A representative number of ectoparasites was randomly removed and stored in 1.5 mL Eppendorf tubes containing 70% ethanol, for subsequent morphological and molecular analysis.

### 2.3 Afoxolaner administration

For treatment, all animals (*n* = 178) were stratified into three weight classes to standardize the dosing, while ensuring the administration of the maximum required dose within each range. The administered dosage (2.5 mg/kg), based on previous studies demonstrating its safety and efficacy in snakes and lizards (Mendoza-Roldan et al., 2023, 2025), was calculated using the upper limit of each body weight class to minimize the risk of underdosing. NexGard® 11.3 mg chewable tablets formulated for small dogs (2–4 kg) were utilized, and the amount of tablet administered was determined proportionally based on the ratio between active ingredient content and total tablet mass (Table 1). Tablets were not homogenized prior to subdivision; instead, they were manually divided into the calculated doses and accurately weighed using an analytical balance (Ohaus AX224; repeatability ±0.1 mg). The drug was administered orally after gently opening the lizard's mouth with the help of forceps.

**Table 1:** Weight classes, calculated afoxolaner doses, and corresponding NexGard® tablet masses, based on the maximum weight in each class.

Number of lizards (*n*)	Body weight range (g)	afoxolaner dose (mg)	NexGard® tablet mass (mg)
39	3.20–6.66	0.0166	0.73
94	6.67–10.12	0.0253	1.12
45	10.13–13.59	0.0340	1.50

Of the 178 subjects, 20 individuals that had high parasitic burdens were selected for physical examination to assess the presence of mites at each time point, and were temporarily isolated in plastic boxes lined with white filter paper to allow collection and quantification of dead mites. Animals were examined pre-treatment (T0) and at multiple post-treatment time points (T1, 24 h; T2, 7 days; T3, 14 days; T4, 28 days) to evaluate their overall health status and clinical signs potentially associated with adverse events. Additionally, at each time point, 30 lizards were randomly selected from their enclosures to evaluate the presence or absence of mites, with different individuals assessed at each sampling. A parallel group of 25 untreated *P. siculus*, housed near the enclosure of *P. raffonei*, was used to monitor potential environmental exposure, given that *P. siculus* naturally harbors Macronyssidae mites (Mendoza-Roldan et al., 2019). Hence, this group allowed for the assessment of the natural environmental load and served to monitor the untreated lizards as a potential reservoir and source of reinfestation for the treated animals. In addition, Lizards of other species, including a free-living *P. muralis* and a housed *Timon lepidus*, observed in proximity to the enclosures, were also examined for mites. To avoid potential bias, no other environmental control measures were applied, ensuring an accurate assessment of afoxolaner's efficacy.

### 2.4 Ectoparasite identification

Genomic DNA (gDNA) from mites was individually extracted using an in-house protocol with phenol/chloroform-isoamyl alcohol (Chomczynski, 1993), to prevent mite destruction and preserve the integrity of the cuticle. Before DNA extraction, ethanol was removed by pipette, and the samples were incubated at 56 °C for 20 min to remove residual ethanol. The same mites were first subjected to molecular analysis and subsequently clarified and mounted on slides in Hoyer's medium for morphological identification using dichotomous keys under a stereomicroscope (Leica Microsystems, MS5, Germany) (Moraza et al., 2009; Gerecke, 2010). Moreover, some specimens were prepared for scanning electron microscopy (SEM) observations. To remove debris from the body surface, the vials containing the specimens in 70% ethanol were cleaned in an ultrasonic bath for 2 min at a frequency of 40 kHz. Subsequently, the specimens were dehydrated through a graded ethanol series, processed with a Baltec CPD030 critical point dryer, and mounted on SEM stubs using double-sided conductive carbon tabs. The samples were then sputter-coated with gold using a Baltec SCD005 apparatus and observed via a Hitachi TM3030 tabletop SEM equipped with a digital camera, at an acceleration voltage of 15 kV.

### 2.5 Molecular screening

Mite's identification was molecularly confirmed in three specimens by conventional PCR (cPCR) using the 18S rRNA gene targeting the V4 region (480 bp) amplified with primers Mite 18S-1F and Mite 18S-1R (Otto and Wilson, 2001). The cPCR 18S rRNA protocol was modified as follows: 95 °C for 10 min initial denaturation, followed by 40 cycles of 94 °C for 35 s, 57 °C for 30 s, 72 °C for 1 min, with a final elongation step at 72 °C for 7 min.

Following this, gDNA of all collected mites was subjected to cPCR for screening Anaplasmataceae using the 16S rRNA gene (∼345 bp, Martin et al., 2005), *Rickettsia* spp. using *gltA* gene (∼400 bp, Labruna et al., 2004) and *Babesia/Theileria* spp., using the 18S rRNA gene (460–520 bp, Gubbels et al., 1999). Negative (i.e., ultra-pure sterile water) and positive controls were included in all PCR runs. The cPCR 16S rRNA Anaplasmataceae (Martin et al., 2005) protocol was modified as follows: 95 °C for 10 min initial denaturation, followed by 35 cycles of 95 °C for 30 s, 60 °C for 30 s, and 72 °C for 30 s, with a final elongation step at 72 °C for 10 min. The cPCR *Rickettsia* (Labruna et al., 2004) protocol was modified as follows: 95 °C for 10 min initial denaturation, followed by 40 cycles of 95 °C for 30 s, 58 °C for 30 s, and 72 °C for 40 s, followed by 72 °C for 7 min for the final elongation. Finally, the cPCR *Babesia/Theileria* (Gubbels et al., 1999) protocol was modified as follows: 95 °C for 10 min initial denaturation, followed by 39 cycles of 95 °C for 30 s, 54 °C for 30 s and 72 °C for 1 min, and then 72 °C for 7 min for the final elongation.

All cPCR products were analyzed on a 2% agarose gel stained with GelRed (VWR International PBI, Milan, Italy) and visualized using a ChemiDoc Touch Gel Imaging System (Bio-Rad, CA, USA). Amplicons were purified enzymatically using Exonuclease I and FastAP Thermosensitive Alkaline Phosphatase, following the manufacturer's protocol (Thermo Fisher Scientific, Waltham, MA, USA), and sequenced in both directions using the same primers as for PCRs. Sequencing was performed with BigDye Terminator v3.1 chemistry on a 3130 Genetic Analyzer (Applied Biosystems, Foster City, CA, USA). Nucleotide sequence analysis was performed with CLC Genomic Workbench v. 22.0.1 (Qiagen Digital Insight, Aarhus, Denmark). Contigs were aligned using CAP3 (Huang and Madan, 1999), and primer sequences were removed from the final assembly. The resulting sequences were compared with those available in the GenBank database by the Basic Local Alignment Search Tool (BLAST) for species identification (https://blast.ncbi.nlm.nih.gov/Blast.cgi).

### 2.6 Phylogenetic analysis

The sequences were individually aligned with those of closely related species available from the GenBank database using the MUSCLE algorithm implemented in MEGA12 software (Kumar et al., 2024). The evolutionary history of *Ophionyssus* spp. was reconstructed using the Maximum Likelihood method. The Tamura-3 parameter model with a proportion of invariant sites (+I) (Tamura, 1992) was used for the 18S rRNA gene. Homologous sequence of 18S rRNA for the tick *I*. *ricinus* (Z74479) was used as an outgroup to root the tree. The phylogenetic tree was supported by 2000 bootstrap replicates.

### 2.7 Statistical analysis of data

The parasitic mite burden was evaluated through descriptive statistical analyses, which were performed using Quantitative Parasitology software (version 3.0; Rózsa et al., 2000). Parameters calculated included prevalence, mean abundance (i.e., the number of mites per the number of examined hosts), and mean intensity (i.e., the number of mites per the number of infested hosts). To evaluate changes over time within the individually and longitudinally tracked group of 20 *P. raffonei* lizards, a Cochran's Q test was used to assess differences in prevalence, and the Friedman test to evaluate changes in mite intensity and abundance. For comparisons between independent groups (e.g., comparing the treated *P. raffonei* against the untreated *P. siculus* indicator group at specific time points), Fisher's Exact Test was used for categorical prevalence data, and the Mann-Whitney *U* test was applied for continuous count data. All inferential statistical analyses and comparisons were performed using GraphPad Prism version 11.0 (GraphPad Software, CA, USA). A *p*-value of <0.05 was considered statistically significant.

## 3 Results

**Fig. 2:**
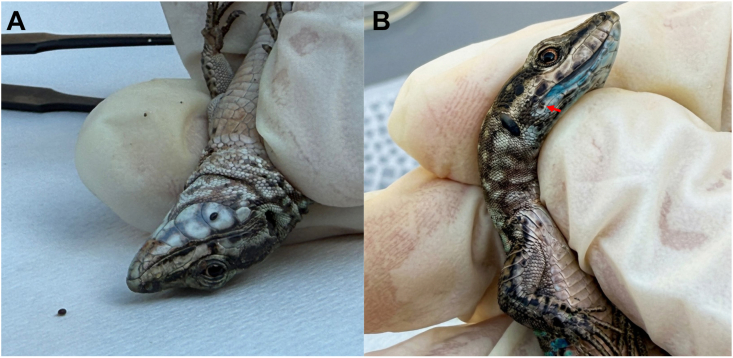
Macronyssidae mites on *Podarcis raffonei*. A) adult mites crawling off a *P. raffonei* lizard during clinical examination; B) mites in the labial commissure of an adult *P. raffonei.*

**Fig. 3:**
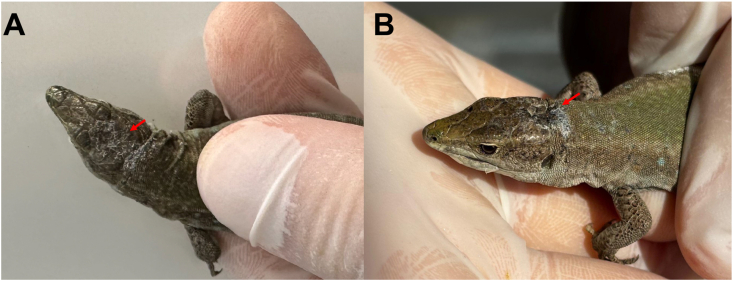
*Podarcis raffonei* lizards presenting dermatological issues during clinical examination. A) dermatitis; B) discoloration.

**Fig. 4:**
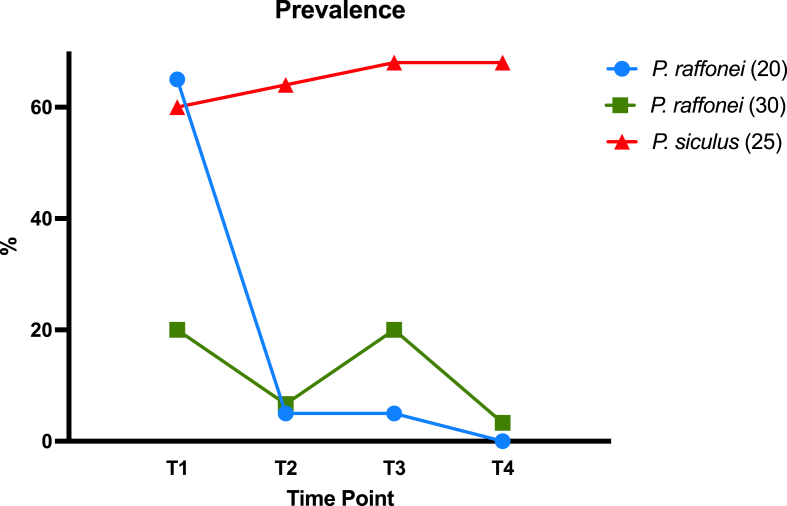
Efficacy of afoxolaner against mite infestation. Mite prevalence (%) is compared across treated lizards: two groups of *P. raffonei* (20 closely monitored lizards and 30 randomly assessed lizards), and an untreated *P. siculus* indicator group, at 24 h (T1), 7 days (T2), 14 days (T3), and 28 days (T4) post-treatment. Treatment yielded almost 100% efficacy by T4, contrasting with the rising prevalence in untreated control.

A total of 178 *P. raffonei* were treated with afoxolaner. Prior to treatment (T0), 90 specimens were infested with mites, with an overall prevalence of 50.6% (95% CI: 43.0–58.1%; Fig. 2A). Among infested lizards, the mean intensity of infestation was 8.97 mites per individual (95% CI: 7.39–10.68%; Fig. 2B), while the mean abundance across the entire population was 4.53 mites per lizard (95% CI: 3.56–5.70%). Four heavily infested individuals, presenting more than 15 mites, showed dermatological alterations including dermatitis (Fig. 3A), and skin discoloration (Fig. 3B). Following the administration of afoxolaner, 20 lizards were examined at each post-treatment time point (T1–T4) to monitor mite infestation (Table 2). The reduction in both the prevalence of infestation and the mean mite abundance across these time points was highly significant (Cochran's Q test, *p* < 0.0001; Friedman test, *p* < 0.0001). At T4 (28 days), no live mites were detected in any treated lizard, although a single dead mite was observed on one individual. Based on the absence of live parasites, treatment efficacy was considered 100% (Fig. 4).

**Table 2:** Prevalence, mean intensity, and mean abundance of mites at four different time points post-treatment with afoxolaner.

Time point	Prevalence	Mean intensity	Mean abundance
T1 (24 h)	65.0 %	3.00	1.95
	(95% CI: 0.408–0.846)	(95% CI: 1.92–4.77)	(95% CI: 1.05–3.35)
T2 (7 days)	5.0 %	6.00	0.30
	(95% CI: 0.001–0.249)	(95% CI: 0.00–0.00)	(95% CI: 0.00–0.90)
T3 (14 days)	5.0 %	5.00	0.25
	(95% CI: 0.001–0.249)	(95% CI: 0.00–0.00)	(95% CI: 0.00–0.75)
T4 (28 days)	0.0 %	-	-
	(95% CI: 0.00–0.168)		
*p*-value	<0.0001∗<	0.0001∗∗<	0.0001∗∗

∗ Statistically significant reduction across time points evaluated via Cochran's Q test. ∗∗ Statistically significant reduction across time points evaluated via the Friedman test.

Additionally, 30 *P. raffonei* were randomly assessed at each post-treatment time point. At 24 h post-treatment (T1), 6 of the 30 lizards remained infested, corresponding to a prevalence of 20% (CI 95%: 7.7–38.6%). By 7 days (T2), prevalence decreased to 6.7% (95% CI: 0.8–22.1%) with only 2 lizards infested. At 14 days (T3), 6 lizards were infested, yielding a prevalence of 20% (95% CI: 7.7–38.6%). By 28 days (T4), infestation had nearly been eradicated, with only 1 lizard positive (3.3%; 95% CI: 0.1–17.2%; Fig. 4).

A parallel control group of 25 untreated *P*. *siculus* was monitored from day 7 (T2) to assess potential environmental mite exposure, statistically confirming the ongoing protective efficacy of the treatment against natural reinfestation. At 24 h post-treatment (T1), prevalence in this untreated group was 60.0% (95% CI: 38.7%–78.9%), similar to the 65% prevalence of the treated individually monitored group. Importantly, at 7 days post-treatment (T2), prevalence in this untreated group was 64.0% (95% CI: 42.5–82.0%), significantly higher than the 6.7% prevalence observed in the treated group (Fisher's Exact Test, *p* < 0.0001). The untreated group presented with a mean intensity of 2.69 mites (95% CI: 1.81–4.31%) per infested individual and a mean abundance of 1.72 (95% CI: 1.00–2.88%) mites per lizard. By 14 days (T3), prevalence in the untreated group increased to 68.0% (95% CI: 46.5–85.1%), which again remained significantly higher than the 20.0% prevalence in the treated lizards (Fisher's Exact Test, *p* = 0.0005). At this T3 time point, the untreated group's mean intensity rose to 5.29 (95% CI: 3.71–8.18%) and mean abundance reached 3.60 (95% CI: 2.24–6.20%; Fig. 4).

**Fig. 5:**
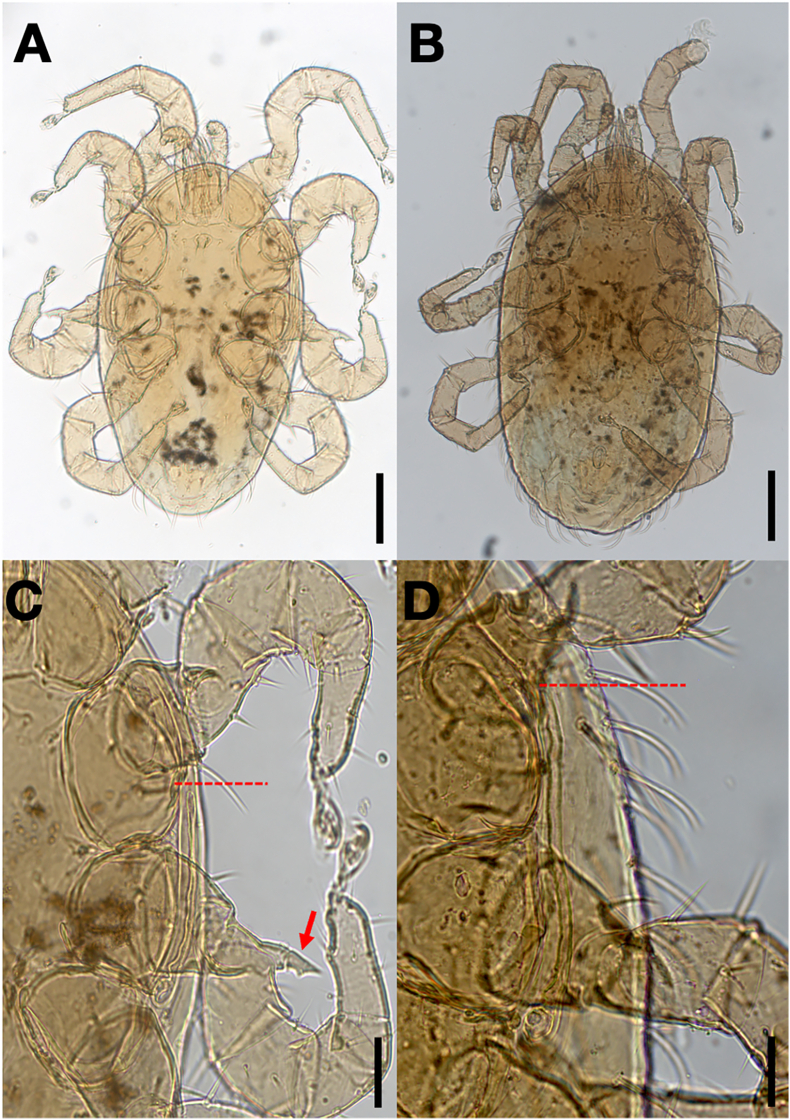
Morphological features of *Ophionyssus lacertinus* under light microscopy. A) Ventral view of adult male; B) ventral view of adult female; C) peritremes extending to posterior margin of coxae II (red dashed line), and femora III with spur-like ventral setae (red arrow) in males; D) peritremes extending to anterior margin of coxae II (red dashed line) in females. Scale bars: A, B 100 μm; C, D 20 μm.

**Fig. 6:**
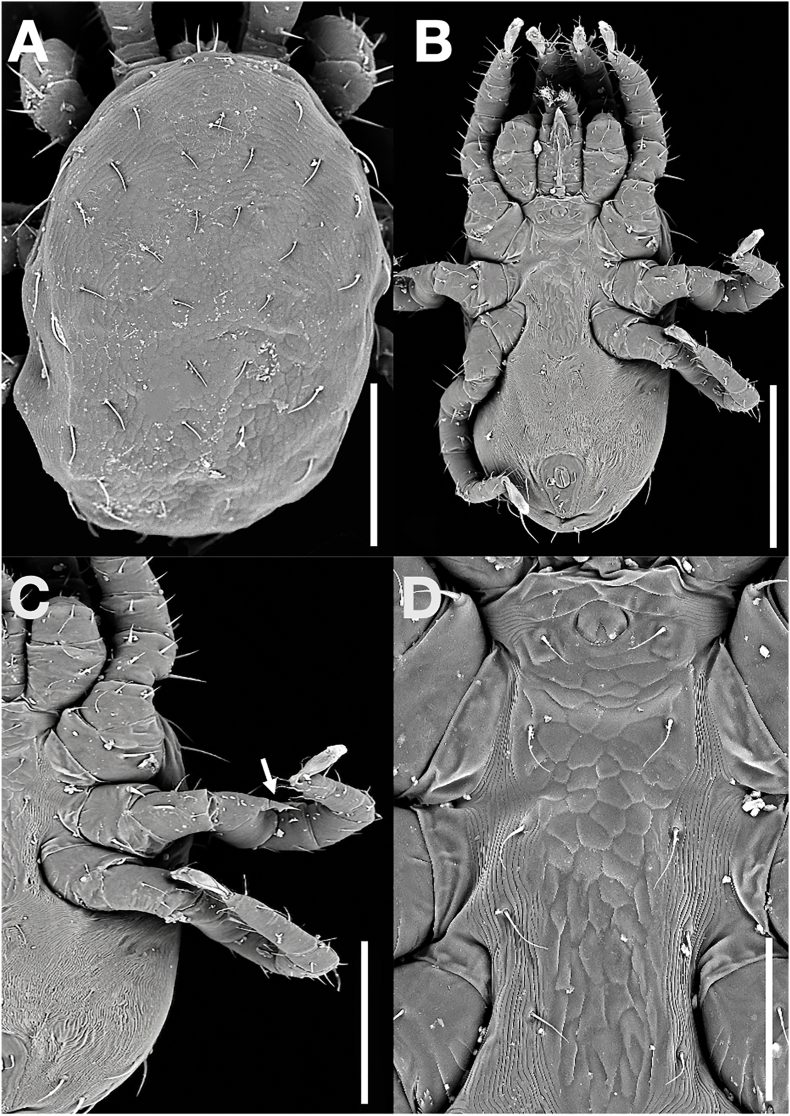
Morphological features of male *Ophionyssus lacertinus* scanning electron microscopy (SEM). A) dorsal view; B) ventral view; C) femora III with spur-like ventral setae (white arrow); D) sternogenital shield with two pair of setae. Scale bars: A, B 100 μm; C 50 μm; D 20 μm.

A total of 48 mites were collected from the examined *P*. *raffonei* lizards and morphologically identified as *Ophionyssus lacertinus* (Berlese, 1892) (Mesostigmata: Macronyssidae) (Fig. 5, Fig. 6). Among these, 24 were males (Fig. 5A), while 9 were non-engorged females (Fig. 5B), 4 were engorged females, and 9 were protonymphs. Two mites - one adult male and one protonymph - were collected from *P. muralis* and also identified as *O. lacertinus*. Additionally, 2 engorged females *O. lacertinus* specimens were recovered from *T. lepidus*. Diagnostic characteristics included male features, such as the dorsal shield features 23 pairs of setae (Fig. 6A), femur III presenting an enlarged, spur-like ventral seta (Figs. 5C, 6B and 6C), and sternogenital shield bearing two pairs of setae (Fig. 6B and D). In females, diagnostics characteristics include an entire dorsal shield with 14 to 15 pairs of setae, peritremes extending to the anterior margin of coxae II (Fig. 5D), and tibia IV with three posterodorsal setae.

**Fig. 7:**
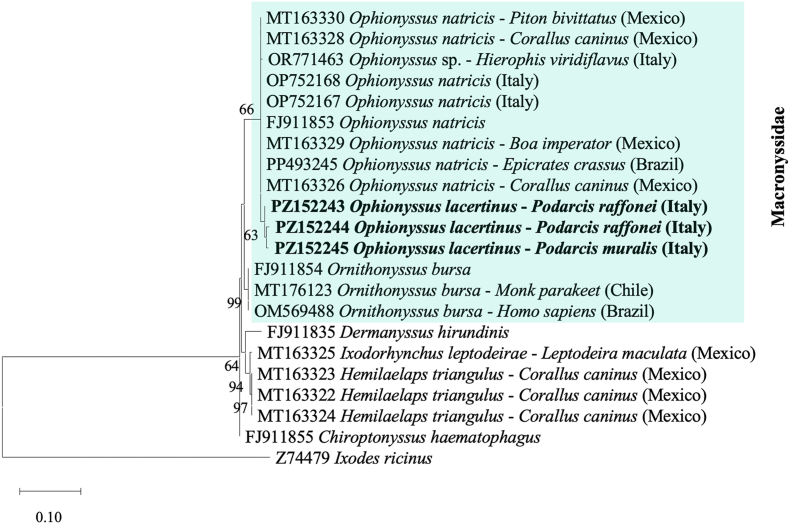
Phylogenetic tree of 18S rRNA gene sequences. Tree was inferred using the Maximum Likelihood method with 2000 bootstrap replicates. The Tamura 3-parameter model with a proportion of invariant sites (+I) was used. Sequences from the present study are shown in bold and are labelled with their GenBank accession numbers, host species, and geographic origin.

Three gDNA from mites from randomly selected mites were subjected to sequencing, and the 18S rRNA results showed 98.39–99.34% identity with *O. natricis* (Accession number OP752168.1). Phylogenetic analysis clustered the sequences obtained from *P. raffonei* and *P*. *muralis* with other *O. natricis* sequences, within the Macronyssidae clade (Fig. 7). Sequences of *O. lacertinus* (18S rRNA) herein generated in this study were deposited in GenBank (AN: PZ152243, PZ152244, PZ152245). Species identification was based primarily on morphological characters, while molecular data provided additional support for placement within the genus *Ophionyssus* and the family Macronyssidae. Furthermore, molecular screening of the examined mites revealed that all samples tested negative for Anaplasmataceae, *Rickettsia* spp. and haemogregarines.

## 4 Discussion

The present study demonstrated that a single oral administration of afoxolaner was effective in controlling Macronyssidae mite infestations in the critically endangered Aeolian wall lizard (*P*. *raffonei*), with complete initial clearance under controlled conditions. Importantly, the treatment was well tolerated in this small lacertid species, with no clinical adverse effects observed at any post-treatment time point, based on daily clinical observations, including assessment of behaviour, feeding activity and general appearance. These findings indicate that this isoxazoline is well tolerated in species with small body size, and furthermore it may represent a promising tool for *ex-situ* conservation efforts before reintroduction (Mendoza-Roldan et al., 2025).

The treatment of *P. raffonei* with afoxolaner resulted in a rapid reduction of mite infestation, with prevalence decreasing from 65.0% to 0% within one month in individually housed lizards. Nonetheless, given the outdoor characteristics of the enclosures and the sympatric occurrence of infested lizards, there is a high likelihood of reinfestation with Macronyssidae mites, which may survive up to three months without feeding in the environment (Orlova et al., 2024). This was observed through the follow-up of the lizards randomly assessed in their outdoor enclosures and with the *P. siculus* population, where mite reinfestation may be perpetuated. Hence, given the confirmed safety of afoxolaner in lacertid lizards, monthly treatment is recommended in this specific high exposure epidemiological scenario. Furthermore, all mites collected from infested lizards were identified as *O. lacertinus*, a species previously described in a Northern population of the wall lizard *P. muralis* in Maastricht, Netherlands (Dietvorst et al., 1980). Moreover, beyond scattered host records, almost nothing is known about the biology of this mite, pathogenic effects, or control (Moraza et al., 2009). Hence, the present study represents the first effort in generating new molecular data for this species, novel morphological description via SEM, as well as the first attempt of pathogen screening. Although all mites were negative for vector-borne pathogens, the dermatological signs recorded in some lizards that had a considerable number of mites further emphasize the need for mite control measures, highlighting the importance of the treatment strategy applied in this study (Hellebuyck et al., 2012; Mendoza-Roldan et al., 2025). Indeed, given that this lizard species has limited documented exposure to mite infestations, preventative treatment with afoxolaner could be considered as part of quarantine protocols in similar settings. Importantly, following the administration, a rapid decline in both prevalence and abundance was observed, which may be consistent with the reported pharmacokinetic persistence of afoxolaner in previous studies conducted in lizards (Mendoza-Roldan et al., 2025) and snakes (Mendoza-Roldan et al., 2023). In captive lizards across multiple families, plasma concentrations were detectable up to 28 days post-treatment, supporting the long-lasting acaricidal activity of the molecule for at least one month (Mendoza-Roldan et al., 2025). Similarly, in snakes naturally infested with *O. natricis*, isoxazoline treatment led to the absence of mites on hosts and in enclosures up to 28 days, supporting monthly administration as a preventive strategy (Mendoza-Roldan et al., 2023). Successful treatment of *O*. *natricis* with afoxolaner was also reported in two Burmese pythons (*Python molurus bivittatus*), with no mites detected 30 days after administration (Gámez et al., 2020).

Additionally, the control group of *P. siculus*, which did not receive afoxolaner treatment, provided an important reference for evaluating the progression of mite infestation, throughout the observation period. This persistence confirms that the reduction observed in the treated group was not due to spontaneous resolution or environmental factors but was attributable to the pharmacological action of afoxolaner. Importantly, although afoxolaner rapidly reduced mite infestations in *P. raffonei*, complete eradication was not achieved within the 28-day follow-up in some specimens from outdoor enclosures. This suggests that untreated *P. siculus*, free-living reptiles and mites persisting in the environment may favor reinfestation (Panarese et al., 2021; Mendoza-Roldan et al., 2025). Moreover, the potential survival of mite life stages in microhabitats such as substrate and enclosures may have contributed to the ongoing infestation (Norval et al., 2020). These findings highlight that parasite control outcomes may be influenced by ecological and management factors, including isolation of the enclosures and the presence of sympatric or nearby host species. A limitation of this study is that the untreated *P. siculus* group was used as an environmental sentinel rather than an untreated control for *P. raffonei*, which may limit the strength of direct comparisons between groups. Compared to other acaricidal treatments reported in reptiles, afoxolaner offers several practical advantages. Traditional pyrethroids require careful rinsing to prevent systemic toxicity (Rockwell and Mitchell, 2019), while Ivermectin, although effective against adult mites, can be toxic to some species and may not affect larval stages (Széll et al., 2001; Hahn et al., 2014; Feng et al., 2023). In contrast, afoxolaner appears to provide a rapid and long-lasting option for controlling mite infestations in lizards, without the need for aggressive environmental treatment.

## 5 Conclusions

Results from this study demonstrate that oral administration of afoxolaner appears effective in controlling mite infestations (e.g., Macronyssidae) in the Critically Endangered Aeolian wall lizard (*P*. *raffonei*), with no observable adverse effects. Pre-treatment assessments revealed a substantial ectoparasite burden, which was sharply reduced within one week of treatment, with prevalence decreasing from 65.0% (i.e., T1) to 5.0% (i.e., T2). No adverse effects were observed in *P. raffonei*, confirming that the dosing was well tolerated in this species. The successful elimination of mites from captive individuals has important implications for conservation programs, as it minimizes the risk of mite infestation and associated pathogens in naïve populations and may contribute to improved health status in reintroduced lizards.

## Funding

The study was partially supported by 10.13039/100020968Boehringer Ingelheim Animal Health (France, Europe). This study was supported by the 10.13039/501100000780European Commission 10.13039/100013288LIFE Programme (Project 101114121 LIFE22-NAT-IT-10.13039/100013288LIFE EOLIZARD. J.A.M.R. and D.O. were partially supported by 10.13039/100016077EU funding within the Next Generation EU-MUR PNRR Extended Partnership initiative on Emerging Infectious Diseases (project no. PE00000007, INF-ACT).

## CRediT authorship contribution statement

**Jairo Alfonso Mendoza-Roldan:** Conceptualization, Data curation, Formal analysis, Funding acquisition, Investigation, Methodology, Project administration, Software, Supervision, Validation, Visualization, Writing – original draft, Writing – review & editing. **Alessia Ricci:** Formal analysis, Investigation, Methodology, Software, Visualization, Writing – original draft, Writing – review & editing. **Benedetta Gambioli:** Data curation, Investigation, Methodology, Visualization, Writing – review & editing. **Katerina Sioumpoura:** Data curation, Investigation, Methodology, Resources, Writing – review & editing. **Antonella Di Palma:** Formal analysis, Investigation, Methodology, Validation, Writing – review & editing. **Gianpaolo Montinaro:** Investigation, Methodology, Resources, Visualization, Writing – review & editing. **Daniele Macale:** Investigation, Methodology, Validation, Visualization, Writing – review & editing. **Leonardo Vignoli:** Data curation, Funding acquisition, Investigation, Methodology, Validation, Visualization, Writing – review & editing. **Frederic Beugnet:** Conceptualization, Funding acquisition, Investigation, Project administration, Resources, Writing – review & editing. **Domenico Otranto:** Funding acquisition, Project administration, Supervision, Visualization, Writing – original draft, Writing – review & editing.

## Declaration of competing interest

Frederic Beugnet is an employee of Boehringer Ingelheim Animal Health (France, Europe). The remaining authors have declared that no competing interests exist.
